# Carotid artery intima-media thickness is closely related to impaired left ventricular function in patients with coronary artery disease: a single-centre, blinded, non-randomized study

**DOI:** 10.1186/1476-7120-12-39

**Published:** 2014-09-29

**Authors:** Kristin Evensen, Sebastian Imre Sarvari, Ole Morten Rønning, Thor Edvardsen, David Russell

**Affiliations:** Department of Neurology, Oslo University Hospital, Rikshopitalet, Postboks 4950 Nydalen, 0424 Oslo, Norway; Department of Cardiology, Oslo University Hospital, Rikshospitalet, Oslo, Norway; Department of Neurology, Medical Division, Akershus University Hospital, Lorenskog, Norway; Institute for Surgical Research, Oslo University Hospital, Rikshospitalet, Oslo, Norway; University of Oslo, Oslo, Norway

**Keywords:** Longitudinal strain, Coronary artery disease, Carotid intima-media thickness, Atherosclerosis, Ultrasound

## Abstract

**Background:**

Atherosclerosis is the underlying cause of the majority of myocardial infarctions and ischemic strokes. Carotid intima-media thickness (IMT) is a surrogate measure of atherosclerotic cardiovascular disease. Left ventricular (LV) function can be accurately assessed by 2D speckle-tracking strain echocardiography (2D-STE). The aim of this study was to assess the relationship between carotid IMT and LV dysfunction assessed by strain echocardiography in patients with coronary artery disease (CAD).

**Methods:**

Thirty-one patients with symptoms of CAD were examined with coronary angiography, cardiac echocardiography and carotid ultrasound. Layer-specific longitudinal strains were assessed from endo-, mid- and epicardium by 2D-STE. LV global longitudinal strain (LVGLS) was averaged from 16 longitudinal LV segments in all 3 layers. LVGLS results were compared with coronary angiography findings in a receiver operating curve (ROC) to determine the cut-off for normal and pathological strain values. The calculated optimal strain value was compared to maximal carotid IMT measurements.

**Results:**

The ROC analysis for strain versus coronary angiography was: area under curve (AUC) = 0.91 (95% CI 0.80 – 1.0), cut-off value for endocardial LVGLS: -16.7%. Further analyses showed that increased carotid IMT correlated with low absolute strain values (p = 0.006) also when adjusted for hypertension, smoking, hyperlipidemia, diabetes and BMI (p = 0.02).

**Conclusions:**

In this study increased carotid IMT values were associated with decreased LV function assessed by strain measurements. These findings support the use of carotid IMT measurements to predict the risk of coronary heart disease.

## Background

The measurement of carotid intima-media thickness (IMT) has long been regarded as a method which can be used to evaluate the presence of generalized atherosclerotic arterial disease [1, 2]. In some reports carotid IMT values have been related to left ventricular (LV) hypertrophy and function [3, 4]. One study of patients with carotid disease showed a possible prediction of minimal cardiovascular resistance in patients with probable CAD [5]. LV function can be assessed with echocardiography using an ejection fraction and wall motion score index, but this method is highly operator dependent. A more reliable method that could detect early LV dysfunction would therefore be of great clinical value. Two-dimensional speckle-tracking echocardiography (2D-STE) is a semi-automated quantitative technique for the assessment of strain in the LV and is a well validated method [6, 7].

Strain is an intrinsic mechanical property which measures myocardial systolic function more accurately and has the ability to detect early pathological changes better than conventional cavity-based echocardiographic measurements [8–10].

The LV wall is a 3-dimensional continuum of myocardial fibers with an inner right-handed helix (endocardium), a middle circular layer and an outer left-handed helix (epicardium). The endocardial layer is the most susceptible to ischemic injury. Layer-specific 2D-STE allows the assessment of LV function in all 3 layers.

Carotid IMT may be used to assess the risk for CAD [1, 2]. Ischemia due to CAD is one of the causes of decreased LV function and there is therefore a possibility that carotid IMT may be used to assess LV function in these patients. The aim of this study was to investigate a possible correlation between IMT and LV function, assessed using strain echocardiography, in patients with CAD.

## Methods

### Patient population

Between September 2010 and January 2012, 31 consecutive patients with suspected CAD were assessed using B-mode ultrasound of the carotid arteries, coronary angiography, cardiac echocardiography and 2D-STE in the endo-, mid-, and epicardium. Inclusion criteria were one of the following: a history of typical or atypical angina or positive ECG stress testing. Exclusion criteria were: age <18 years, acute coronary syndrome (ST-elevation or non-ST elevation myocardial infarction) in the preceding 3 months, concomitant significant disease or non-cardiological therapy which could affect cardiac remodelling or function, left bundle-branch block, severe valvular dysfunction, atrial fibrillation, sustained severe arrhythmia, or any condition which interfered with the patients` ability to comply.

The study was approved by the Regional Ethic Committee (REK), Norway and subjects provided written informed consent.

### Carotid ultrasound

**Figure 1:**
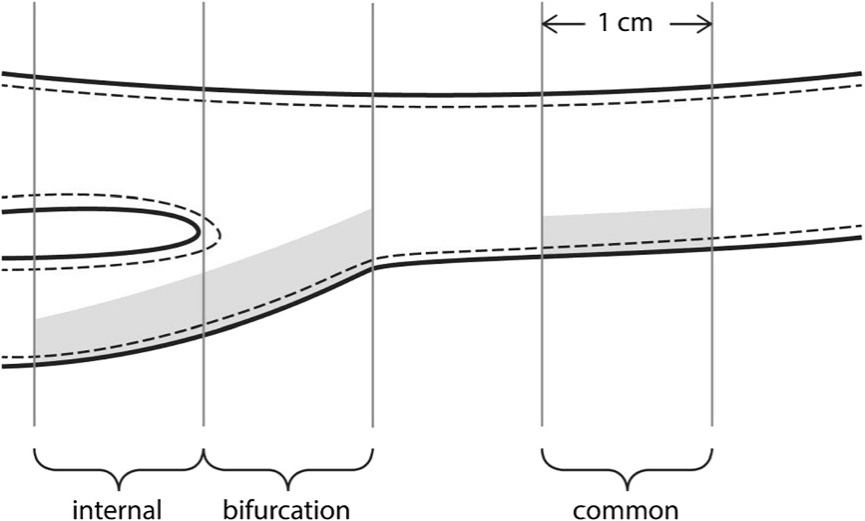
**Drawing of a carotid artery showing the location (in grey) for the measurements of intima-media thickness (continuous-stippled line) in our study.**

**Figure 2:**
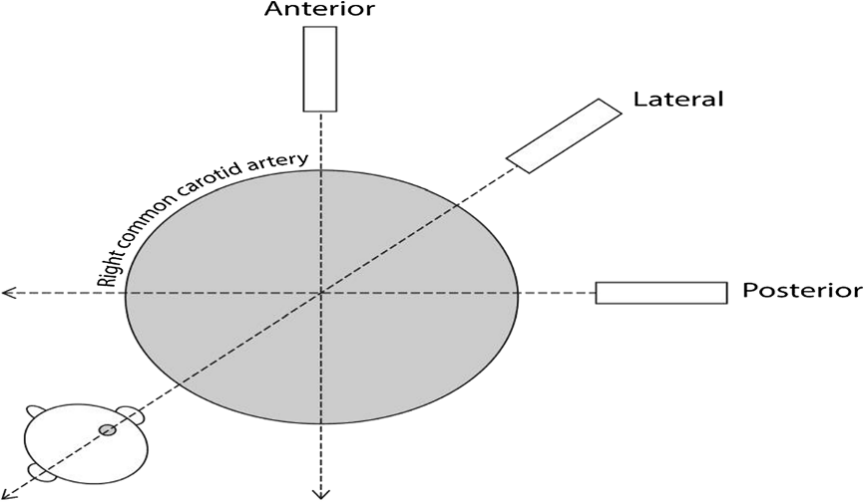
**Drawing of the right common carotid artery showing the probe orientations for the ultrasound examinations.**

Patients were examined in the supine position with the head angled approximately 45 degrees towards the contralateral side. IMT measurements were synchronized with the QRS complex on the ECG. Measurements were made in each carotid artery at the peak of the R-wave where the lumen is widest. IMT was defined as the distance between the lumen-intima and media-adventitia borders of the vessel identified as a double line pattern in a longitudinal image [11]. IMT was measured in the carotid arteries on both sides in 3 segments (Figures 1 and 2):

1. In the far wall of the common carotid artery (CCA) 1 cm proximal to the bifurcation over an area of 1 cm and in 3 different projections: lateral, posterior and anterior.
2. In the far wall of the carotid bifurcations (BIF) over an area of 1 cm in the lateral position.
3. In the far wall of the proximal internal carotid arteries (ICA) immediately distal to the bifurcation over an area of 1 cm in the lateral projection.

This gave a total of 10 IMT measurements in each patient. We used the highest maximum IMT in the CCA, BIF and ICA from either side of the neck for further analyses. Plaque diameter was included in the IMT measurements if present in the areas of interest. The carotid ultrasound examinations were carried out according to the American Society of Echocardiography guidelines [12], using a General Electric Vivid 7 ultrasound instrumentation with a linear M12L probe (14 MHZ) (General Electric, Horten, Norway).

### Coronary angiography

All patients underwent coronary angiography. The assessment of CAD was performed by visual estimates in each single stenosis with multiple projections, avoiding overlap of side branches and foreshortening of relevant coronary stenoses. Coronary occlusion was defined as TIMI flow 0 or 1, while significant coronary artery stenosis was considered as ≥50% reduction of vessel diameter in at least one major coronary artery.

### Two-dimensional echocardiography

Echocardiographic studies were performed with a commercially available system (ARTIDA, Toshiba Medical Systems Corporation, Tokyo, Japan). Digital routine grey scale 2D cine loops from 3 consecutive heart beats were obtained at end-expiratory apnoea from 3 standard apical views (4-chamber, 2-chamber, and long-axis). Frame rates were 47 ± 5 Hz for the grey scale imaging. The echocardiographic data were analysed blinded for all clinical information.

### Two-dimensional speckle-tracking strain echocardiography

**Figure 3:**
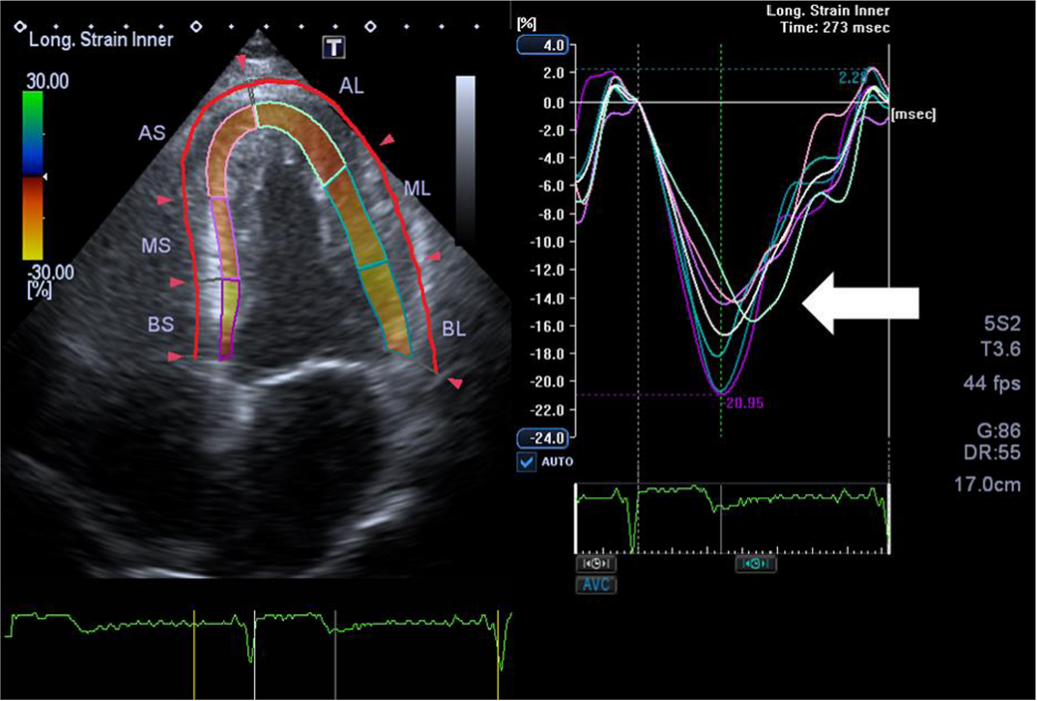
**Endocardial longitudinal strain study of a patient with significant coronary artery disease.** The automatic strain analysis in a patient with angina and significant left anterior descending (LAD) artery stenosis. The apical four-chamber view showes reduced colour coded subendocardial strain values segments supplied by the left LAD artery. Colour-coding from yellow to green indicates strain from +30% to -30%. Yellow = normal strain. Brown = areas with reduced strain. On the right strain curves for the 6 subendocardial segments are presented. The white arrow shows reduced strain values of – 14% in the curves representing the segments supplied by the LAD artery. AL = apicolateral; AS = apicoseptal; BL = basolateral; BS = basoseptal; ML = midlateral; MS = midseptal.

Myocardial function by strain was evaluated on a frame-by-frame basis by the automatic tracking of acoustic markers (speckles) throughout the cardiac cycle. The endocardial borders were traced in the end-systolic frame of the 2D images from the 3 apical views for analyses of longitudinal endocardial, mid-myocardial and epicardial strain (Figure 3). Peak systolic longitudinal strain from 3 layers was assessed using off-line software (Toshiba Medical Systems Corporation, Tokyo, Japan) in 16 longitudinal segments. All segmental values were averaged to global longitudinal strain (GLS). Segments that failed to track properly were manually adjusted by the operator. Any segments that subsequently failed to track were excluded.

### Statistical analysis

Data were presented as mean ± standard deviation (SD), numbers and percentages respectively. Student`s t-test (continuous variables) was used to determine differences between two groups. We used multiple linear regressions to determine the relationship of carotid IMT with hypertension, smoking, hyperlipidemia, diabetes and BMI. (SPSS version 18, SPSS Inc, Chicago, Ill). The areas under the receiver operating characteristics (ROC) curves (AUC) were calculated for endocardial, mid-myocardial, epicardial and carotid IMT. The values closest to the upper left corner of the ROC curves determined optimal sensitivity and specificity for the ability of endocardial, mid-myocardial, epicardial and carotid IMT to predict the presence of significant CAD. Sensitivity and specificity was calculated using openepi.com (Open Source Epidemiologic Statistics for Public Health, Version 2.3.1.).

## Results

Thirty- one patients took part in the study. Two patients were excluded due to poor echocardiographic image quality. Strain analyses were therefore performed in 29 patients.

The age of the patients ranged from 53 to 75 years. There were 81% males in the study. Baseline characteristics of the patients are shown in Table 1.

**Table 1:** **Patient characteristics**

Number of patients, n	31
Age (range)	64 (53–75)
Males, n (%)	25 (81)
TIA and ischemic stroke, n (%)	0
Heart attack (former), n (%)	5 (16)
Diabetes, n (%)	5 (16)
Hypertension, n (%)	17 (55)
Hyperlipidemia, n (%)	12 (39)
Smoking, n (%)	5 (16)
BMI	28 ± 4

TIA = transient ischemic attack; BMI = body mass index.

A total of 20 (65%) patients had significant CAD (≥50% stenosis). Eight of these patients had an occlusion of a major coronary artery) and 11 (35%) had a non-significant CAD (<50% stenosis). There was a significant difference in endocardial and mid-myocardial LVGLS between the patients with significant coronary stenosis and the patients with non-significant coronary stenosis. Layer-specific LVGLS compared to the coronary angiography findings are shown in Table 2.

**Table 2:** **Layer-specific global longitudinal strain of the left ventricle compared to coronary angiography findings**

	< 50% stenosis (n = 11)	≥ 50% stenosis (n = 18)	p-value
Endocardial LVGLS	- 19.5 ± 2.2	-15.0 ± 2.7	0.001
Midmyocardial LVGLS	-15.4 ± 1.6	-13.1 ± 2.6	0.011
Epicardial LVGLS	-12.8 ± 1.9	-11.6 ± 2.2	0.169

Left ventricular global strain (LVGLS) is in mean ± SD.

Left ventricular Ejection Fraction (LVEF) was: mean ± SD; 61% ± 6% for the total group of patients. Patients with normal endocardial strain had EF values of: mean ± SD; 62% ± 6.0% and patients with reduced endocardial strain mean ± SD; 58% ± 4.8%. This difference was not significant (p = 0.091).

**Figure 4:**
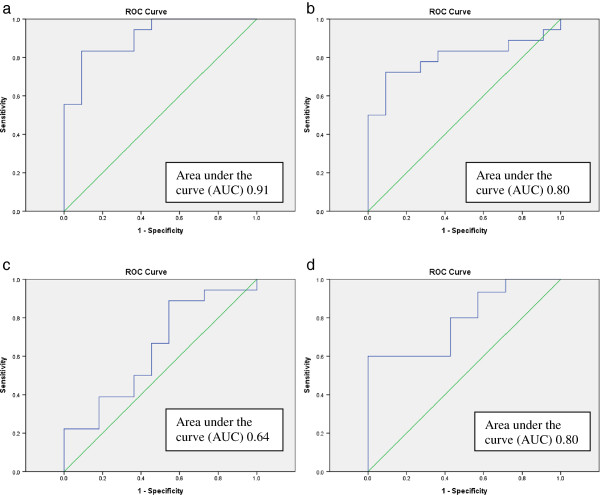
**ROC curves for coronary angiography and carotid IMT versus ventricular strain. a**: Coronary angiography versus endocardial 2D-STE. **b**: Coronary angiography versus mid-myocardial 2D-STE. **c**: Coronary angiography versus epicardial 2D-STE. **d**: Average maximum Carotid IMT versus endocardial 2D-STE.

Figures 4a-d shows ROC analyses of layer specific strain parameters and the ability of IMT measurements to identify patients with significant CAD. Maximum carotid IMT ranged between 1 mm and 2.88 mm for the whole group of patients including carotid plaques in the IMT measurements. For the group with normal endocardial strain values, defined by the cut-off strain value of -16.7, carotid IMT was 1.2 mm ± 0.2 (mean ± SD) and for the group with reduced endocardial strain values the carotid IMT value was 1.7 mm ± 0.5 (mean ± SD). Independent samples t-test comparing the two groups was statistically significant (p = 0.006). There was significant correlation between endocardial LVGLS and maximum carotid IMT (p = 0.006). The results of linear regression analyses which incorporated adjustment for known risk factors of atherosclerosis (hypertension, smoking, hyperlipidemia, diabetes and BMI) were made and showed significant results (p = 0.02).

## Discussion

In this study we found a significant correlation between increased carotid IMT and LV dysfunction. We also found associations between significant CAD assessed by coronary angiography and reduced endocardial and mid-myocardial function assessed by 2D speckle-tracking strain echocardiography.

The cut-off value for endocardial strain was approximately -16.7% whereas normal strain values are approximately -20% [13]. We had no gold standard with regard to normal and abnormal 2D-STE values which could be used for the ROC curves comparing carotid IMT versus 2D-STE. We therefore calculated a cut-off value for pathological endocardial strain measurements. We found affection of LV function in the endocardial and midmyocardial layers but not in the epicardial layer. This finding is in accordance with the fact that, reduced myocardial function usually starts in the endocardial layer and this layer is the most susceptible layer for ischemic injury [14].

There are few studies investigating the association between carotid disease and early left ventricular injury but there are some findings which correspond to the present study. The correlation between LV dysfunction and increased carotid IMT was found in one study of individuals free of clinical CAD, where greater carotid IMT was associated with changes of myocardial strain, measured by tagged MRI [15]. Mizuguchi et al. found that LV relaxation measured by 2D-STE was associated with carotid arterial atherosclerosis in particular related to sclerosis, measured by carotid arterial stiffness. They did not find this correlation for carotid IMT and LV strain [16].

The assessment of LV function is challenging due to its very complex deformation pattern. All imaging modalities have shortcomings in this regard. However, although myocardial strain measures are only a component of LV function, it has been demonstrated to be an accurate and reproducible measure in several studies [6, 7].

All patients in our study had symptoms of CAD or risk factors for atherosclerosis. It is known that structural and functional changes in the carotid arteries occur with normal aging. However, the presence of cardiovascular risk factors accelerate these changes [17–19].

It is well known that carotid intima-media thickness and plaques have different pathophysiological mechanisms. However, both carotid IMT and plaques are well-known indicators of increased risk for cerebro- and cardiovascular disease [20, 21]. We therefore included the diameter of the small number of plaques when detected in the area of interest.

In a study of patients with hypercholesterolemia and preserved LVEF, 1 year treatment with a statin improved regional LV systolic and diastolic function measured by strain [22]. Carotid stiffness was lower after statin therapy, but there was no difference in carotid IMT before and after treatment with a statin. This is probably due to the fact that the annual rate of IMT progression is lower than the resolution of carotid ultrasound (≈0.3 mm). The presence of increased IMT may lead to detection of very early cardiac dysfunction which may benefit from cardiovascular risk management.

The results of this study support the few previous studies which have examined a possible association between carotid IMT and LV strain. B-mode ultrasound of the carotid artery is a non-invasive, inexpensive and widely used examination and may therefore be an additional appropriate tool for the assessment of possible CAD. An ultrasound examination of the carotid arteries is mandatory in all cases with cerebrovascular disease. A more detailed examination which includes IMT measurements in patients with cardiovascular risk factors may therefore be of value when estimating the risk of subclinical coronary artery disease.

## Conclusion

In this study increased carotid IMT values were associated with decreased left ventricular function. These findings support the use of carotid IMT measurements to predict the risk of coronary heart disease.

## Abbreviations

AUC: Area under the curve
BIF: Bifurcation
BMI: Body mass index
CAD: Coronary artery disease
CCA: Common carotid artery
CI: Confidence interval
ECG: Electrocardiography
GLS: Global longitudinal strain
ICA: Internal carotid artery
IMT: Intima-media thickness
LV: Left ventricle
LVGLS: Left ventricular global longitudinal strain
MRI: Magnetic resonance imaging
NPV: Negative predictive value
PPV: Positive predictive value
ROC: Receiver operating characteristics
SD: Standard deviation
TIA: Transitoric ischemic attack
2D-STE: Two-dimensional speckle- tracking strain echocardiography.
